# Acromion fracture and lateral angle of the scapula spine: Case report and literature review

**DOI:** 10.1016/j.ijscr.2019.06.036

**Published:** 2019-06-22

**Authors:** Sara Ribeiro Nunes, Marcos Rassi Fernandes Filho, Marcos Rassi Fernandes

**Affiliations:** a Goiás Federal University, Brazil; b Student of High School

**Keywords:** Shoulder injuries, Fracture fixation, Shoulder blade, Acromion, Diagnosis by image

## Abstract

**Introduction:**

Scapular fractures are uncommon and correspond to 0.5–1% of all body fractures. The study objective was to present a rare case report of a fracture (lateral acromion and angle of the scapula spine) and review of the literature on which will greatly contribute to diagnosis and treatment.

**Presentation of case:**

A 74-year-old female patient with comminuted fracture of the acromial process and lateral angle of the spine of the scapula with subacromial space. The patient underwent surgical treatment with open reduction and use of blocked plaque, two plain Steinmann wires 2.0 and local bone graft.

**Discussion:**

Segmental fracture of acromion and spine of the scapula needs early diagnosis for the proper treatment. In our case report, the patient presented fracture of the acromion and lateral angle of the spine of the scapula, classified as Ogawa 1 and 3; Kunt 3; and AO A1, respectively.

**Conclusion:**

Segmental fracture of the acromion and lateral angle of the spine of the scapula with subacromial space reduction requires surgical intervention in order to avoid secondary complications. Early diagnosis favors better prognosis.

## Highlights

• Fractures of the scapula are rare, especially in the lateral angle.
• Computed tomography helps to classify fracture types.
• Fractures with risk of bipartition is indicated surgical fixation.

## 1 Introduction

Fractures in the scapula are considered uncommon in traumatology, representing 3%–5% of the fractures in the shoulder region [1,2], the most common ones occur in the body, neck, glenoid and acromion, respectively [[2], [3], [4], [5]]. The fracture of the spine of the scapula, especially in its lateral angle, is rare, and often, this anatomical location is confused with the acromion itself, although the latter is defined from its anterior portion to the acromial [6].

In addition, the association between acromial fracture and the lateral angle of the spine of the scapula has its frequency unknown and presents as probable mechanism the direct trauma of high energy in the upper region of the shoulder [2,3,7,8]. The article was to report the case of an association of fracture of acromion and lateral angle of the spine, as well as treatment and review of the literature. This report is in accordance with the criteria established by SCARE [9].

## 2 Presentation of case

A 74-year-old female patient suffered direct left shoulder trauma by fall from own height, with complaints of pain and functional disability. She had diabetes mellitus and systemic arterial hypertension as comorbidities.

**Fig. 1:**
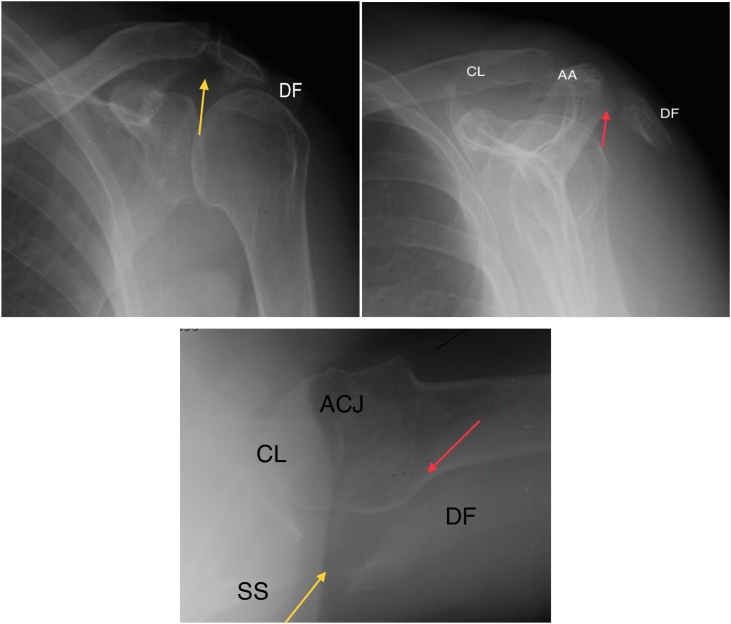
Left shoulder x-rays. a) AP view, yellow arrow: displaced fracture of lateral angle of the scapular spine; b) outlet view, red arrow: displaced acromion fracture; c) axillary view, yellow and red arrows: the same as figure a and b; CL- clavicle; AA- anterior acromion; DF- displaced fragment; SS- spine of the scapula; ACJ- acromioclavicular joint.

**Fig. 2:**
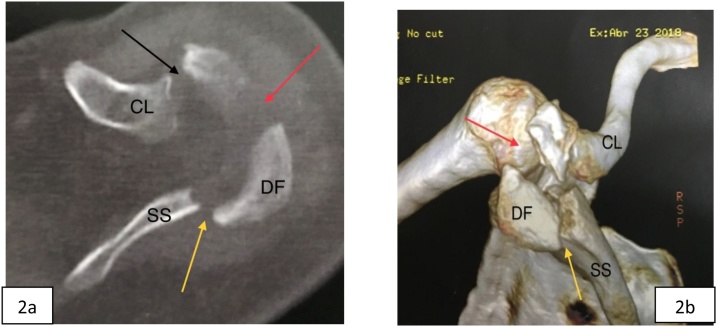
Left shoulder CT (a) with 3D reconstruction (b). yellow arrow: displaced fracture of lateral angle of the scapular spine; red arrow: displaced acromion fracture; black arrow: acromioclavicular joint; CL- clavicle; DF- displaced fragment; SS- spine of the scapula.

On physical examination, were observed whole skin, diffuse edema and limited movements of the left shoulder. The neurovascular status of the limb was preserved. Simple radiographs of the shoulder (series trauma) were performed in AP, profile and axillary profile (Fig. 1), in initial consultation at the medical center. The patient was immobilized with simple sling because of suspected fracture in the scapula and she was oriented to look for a shoulder specialist, which only happened 16 days after the fall. A computerized tomography scan of the shoulder with three-dimensional reconstruction (Fig. 2) was requested, however the diagnosis was only confirmed 37 days after the trauma, due to delayed patient return with the exam results: deviated fractures of acromion (Ogawa-1; Hunt-3; AO-A1) [6,10,11] and lateral angle of the spine of the scapula (Ogawa 3; Hunt 3) [6,10].

After the analysis of the imaging exams, the decision was made to do a surgical intervention, which occurred only after two and a half months from the date of the trauma due to delay in returning to the hospital with health service clearance. Therefore, the surgery happened already in a phase of delay of fracture consolidation.

**Fig. 3:**
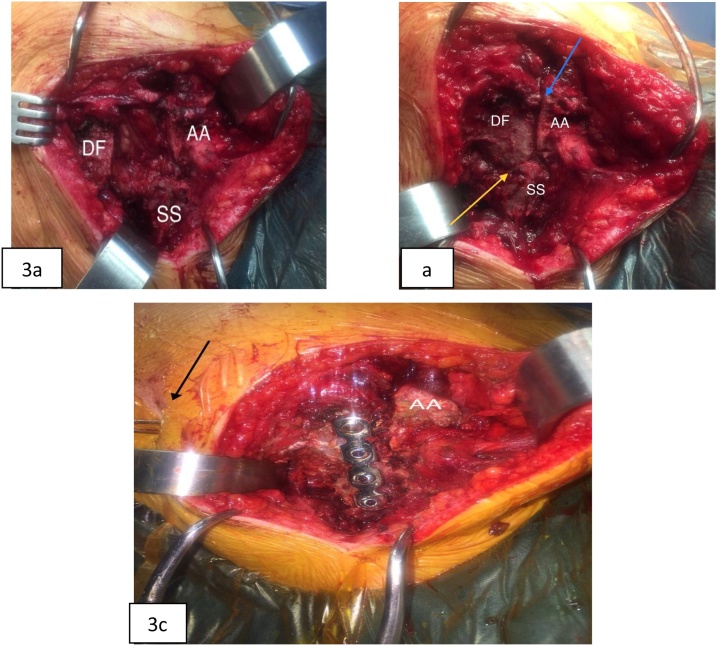
Surgical Procedure: a) intra-operative displaced fractures image; b) open reduction of both fractures, yellow arrow: displaced fracture of lateral angle of the scapular spine; blue arrow: displaced acromion fracture; c) osteosynthesis of both fractures with blocked plate and screws + steimann wires (black arrow); AA- anterior acromion; SS- spine of the scapula; DF- displaced fragment.

**Fig. 4:**
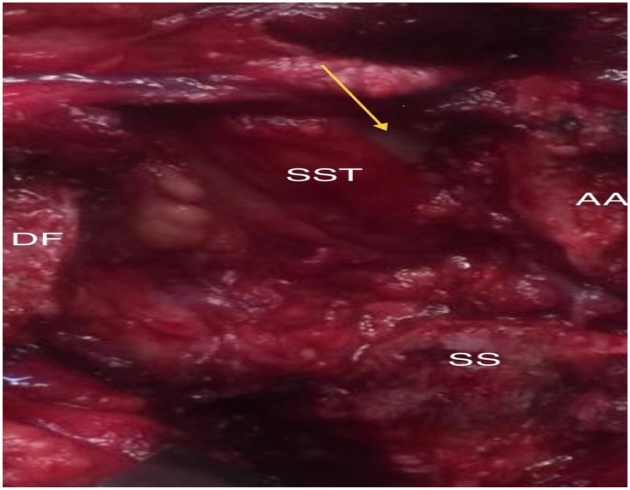
Intraoperative rotator cuff tear (yellow arrow). AA- anterior acromion; SS- spine of the scapula; DF- displaced fragment; SST- supraspinatus tendon.

**Fig. 5:**
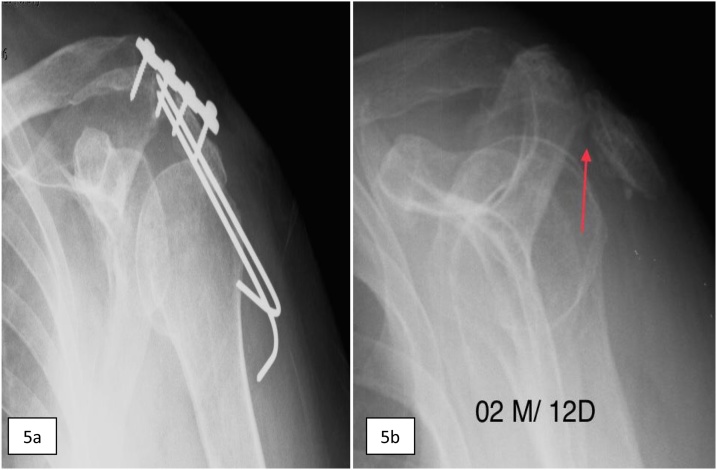
Post-operative left shoulder x-rays. a) AP view (immediate P.O.); b) outlet view, red arrow: acromion fracture after removal of osteosynthesis material.

An open reduction was achieved by post-lateral access between the muscles trapezius and deltoid, addressing segmental fractures. It was noticed that there was a complete chronic rupture of the associated rotator cuff (Fig. 3). The fracture of the lateral angle of spine of the scapula was fixed with locked plate of mini-fragments with four cortical screws, while the fracture of acromion with two Steinmann yarns 2.0, from posterior to anterior, because of the fragility of the bone and risk of posterior fragment split (Fig. 4). Was also withdrawn bone graft and placed on the upper edge of the acromion fracture. Fractures were stabilized, although the wires do not offer rigid fixation. The patient was immobilized with Velpeaux sling. A radiograph in AP on post-surgery was made (Fig. 5a).

Following the surgical procedure, periodic radiographic examinations were performed to follow the bone healing of the fractures (Fig. 5b). Steimann’s wires showed loose with 2 months postoperative, which were removed, as was the locked plate. The patient remained under medical care, in the use of immobilization in the upper limb, until complete fracture consolidation. The amplitude of shoulder movements in abduction, anterior elevation and rotations was normal.

## 3 Discussion

Anatomically, the scapula located at the posterosuperior border of the thorax and attached by several muscles to the gradil arcs, plays an important role in the biomechanics of the upper limb [3]. On its posterior face we have the scapula spine, which can be easily palpated, which ends in an apophysis, acromion, on the inner edge of which there is a joint facet for the clavicle [3,8].

The fracture of the scapula presents an index of 0.5–1% of all fractures of the body, and therefore are considered uncommon [1,7,[12], [13], [14]] and this can be explained by its great mobility and protection by several layers muscle cells [1,3,12]. Among these, the fracture of the acromion and the spine of the scapula represent 7% and 6%, respectively [13,15].

There are three classifications related to acromio fractures in the literature [6,10,11]. In our case report, this fracture was classified as Ogawa [6] type 1; Kunt [10] type 3 and AO [11] type A1, respectively, similar to the finding of Cicekli et al. [16]. Mardy et al. [17], on the other hand, presented a fracture of acromion only classified as Kuhn type 3.

The "axillary profile" radiographic evaluation is the main incidence to be performed to diagnose fractures in these locations. Computed tomography is a more detailed imaging exam, therefore, essential to verify such bone lesions, and when performed with three-dimensional reconstruction, identification becomes even easier [3,17]. In the studies by Hil et al. [18] and Cicekli et al. [16], radiographic and computed tomography examinations also confirmed the fracture of the acromion and made it possible to classify them.

Ultrasonography and magnetic resonance imaging are used for the assessment of associated soft tissue injuries in the affected region [19], which in our view was not justified at the time of the trauma, due to the complexity of the clinical picture presented for the treatment.

When analyzing the imaging examinations, it was decided to do open reduction, because the fractures were deviated with reduction of the subacromial space, which prevented the secondary impact and favored an early rehabilitation. Surgical intervention in bone lesions on this topography minimizes the possible complications when compared to non-surgical treatment [18].

During the surgical approach it was decided by the use of locked mini-fragment plate in the lateral angle fracture of the spine of the scapula and two smooth Steinmann wires 2.0 for the fracture of the acromio. In the study on acromial fracture fixation surgery [19], the author disagreed with the use of plain wires because they did not perform compression necessary to promote bone consolidation, which we agreed upon, however, in this case, there was a risk of split the fragment by time of evolution and intense bone fragility.

In the present report, a stable fixation was observed during the surgical procedure, however, as previously stated, the patient already presented for surgery, in a period of evolution of more than two months, which already characterized a delay of consolidation.

Literature is scarce with regard to lateral angle spine fracture of the scapula and acromio [7,9,12,13,[16], [17], [18], [19]], and are mostly individualized case reports. To our knowledge, this case report is the first to show these concomitant fractures, with pre and postoperative examinations, treatment and evolution, associated with a review of the literature on the subject.

## 4 Conclusion

Segmental fracture of the acromion and lateral angle of the spine of the scapula with reduction of the subacromial space requires surgical intervention. Early diagnosis favors a better prognosis.

## Conflicts of interest

The authors deny a conflict of interest for the publication of this article.

## Sources of funding

The authors declare that sponsors were not involved in the study.

## Ethical approval

The Case Report follows ethical standards for human research. The hospital in which the patient was treated became aware of the whole procedure and of the intention to publish the protocol of care in which the patient was submitted.

The patient was informed about the entire surgical procedure, with the risks and benefits and agreed by sgning the free and informed consent term in which he declares his authorization for the disclosure of the case report in this way we declare that the study was in accordance with ethical criteria.

## Consent

Written informed consent was obtained from the patient for publication of this case report and accompanying images. A copy of the written consent is available for review by the Editor-in-Chief of this journal on request.

## Author’s contribution

Marcos Rassi Fernandes (MRF) contributed to perform the operation, to collect, to analyze data, to draft and revise the manuscript, to create illustration and to approve for publishing.

Sara Ribeiro Nunes (SRN) and Marcos Rassi Fernandes Filho (MRFF) contributed to data collect, to draft and revise the manuscript and to approve for publishing.

## Registration of research studies

Does not present a search record because it is a case report.

## Guarantor

Marcos Rassi Fernandes (MRF) and Sara Ribeiro Nunes (SRN).

## Provenance and peer review

Not commissioned, externally peer-reviewed.
